# Recent Advance in Tumor Microenvironment-Based Stimuli-Responsive Nanoscale Drug Delivery and Imaging Platform

**DOI:** 10.3389/fphar.2022.929854

**Published:** 2022-07-22

**Authors:** Fengzhi Cui, Jianhua Liu, Siwen Pang, Bo Li

**Affiliations:** Department of Radiology, The Second Hospital of Jilin University, Changchun, China

**Keywords:** stimuli-responsive nanoscale drug delivery, imaging platform, nanomaterials, TME, cancer therapy

## Abstract

The tumor microenvironment (TME) plays an important role in the development, progression, and metastasis of cancer, and the extremely crucial feature is hypoxic and acidic. Cancer-associated fibroblasts (CAFs), extracellular matrix (ECM), mesenchymal cells, blood vessels, and interstitial fluid are widely recognized as fundamentally crucial hallmarks for TME. As nanotechnology briskly boomed, the nanoscale drug delivery and imaging platform (NDDIP) emerged and has attracted intensive attention. Based on main characteristics of TME, NDDIP can be classified into pH-sensitive delivery and imaging platforms, enzyme-sensitive delivery and imaging platforms, thermo-sensitive delivery and imaging platforms, redox-sensitive delivery and imaging platforms, and light-sensitive delivery and imaging platforms. Furthermore, imageology is one of the significant procedures for disease detection, image-guided drug delivery, and efficacy assessment, including magnetic resonance imaging (MRI), computed tomography (CT), ultrasound (US), and fluorescence imaging. Therefore, the stimuli-responsive NDDIP will be a versatile and practicable tumor disease diagnostic procedure and efficacy evaluation tool. In this review article, we mainly introduce the characteristics of TME and summarize the progress of multitudinous NDDIP as well as their applications.

## Introduction

Cancer is the most common cause of death worldwide threatening human health seriously. According to cancer statistics in 2021, approximately nearly one million new cancer deaths and six hundred thousand cancer deaths were reported. The incidence and mortality rates of cancer have continuously increased for the past few years (Siegel et al., 2021). Despite traditional therapies for cancer, surgery, radiotherapy, chemotherapy, and all kinds of other therapy have made significant and remarkable achievements. These also lead to many systemic side effects such as 1) although cancer resection greatly prolongs patients’ survival time, it reduces the survival quality (Nam and Shin, 2021); 2) chemotherapy drug is known to have adverse side effects such as drug resistance, nonspecific toxicity, and short circulating half-life (YAN et al., 2020); 3) in view of the conventional radiotherapy with many restrictions lead to long-term destruction of normal organ functions as well as the immune system (Zhang et al., 2019). In consequence, novel and efficient therapeutic methods (Naidoo and Chuturgoon, 2021) with fewer side effects, high efficiency, and low toxicity still urgently need to be explored.

With the remarkable and rapid progress of nanotechnology, the nanomaterial is gradually emerging and has become a global research spotlight (Bhutta et al., 2021). Based on their unique advantages, such as extremely small size, high stability performance, and favourable biocompatibility properties, nanomaterials have attracted intensive attention, giving rise to the novel bellwether as nanomedicine, especially in noninvasive diagnosis, precise therapy (Jiang et al., 2021), and prevention of cancer (Xiang et al., 2020; El-Sayed and Kamel, 2020; Wang et al., 2021; HE et al., 2015). Nanomaterials can be produced into a variety of spatial structures, morphology, forms, and surface modification based on their intrinsic thermal, electrical, magnetic, and optical physical properties. Nanomaterial is widely used as a theranostic agent in PTT (photothermal therapy) (Huang et al., 2021), PDT (photodynamic therapy) (Bharathiraja et al., 2018; Wu et al., 2021), CDT (chemodynamic therapy) (Wang et al., 2022; Yang et al., 2022), and NDDS (Bai et al., 2021; Foglietta et al., 2021) to overcome the limitations of standard cancer treatments.

A variety of nanomaterials have attracted a lot of research attention as diagnostic and therapeutic tools to promote the exploration of nanomedical cancer therapy. As a typical paradigm, Liu et al. have synthesized NaYbF_4_:Tm@NaGdF_4_:Yb-PVP nanoparticles to serve as the most attractive candidates to achieve UCL/MRI/CT tri-modal imaging and improve the detected rate of early-stage tumor (Wang et al., 2017). In another representative work, Li et al. constructed a magnetic composite nanoplatform using cancer PTT, by coating with the PDA on the surface of Fe_3_O_4_ nanoparticles. Such a well-designed composite therapeutic nanoparticles exert its cancer therapeutic effect, promoting the application of nanomedical in cancer therapy (Li et al., 2020). In a significant work in 2018, Liu et al. further demonstrated the protective effects of CFNs, integrated PDT, CDT, PTT, and MRI imaging capabilities, achieving synergistic cancer treatment. The “all-in-one” nanoplatform has tremendous potential in cancer theranostics (Liu et al., 2018). In light of this, Deng et al. prepared targeted multifunctional UCHNs-PAMA nanocarriers that offered the enhanced T_1_-weighted MRI, CT, and upconverting luminescence (UCL) imaging. Meanwhile, the nanoparticle-loaded Pt(VI) has effective inhibition for combinational therapy of cancer *via* the *in vitro* and *in vivo* experiments confirmed. In consequence, UCHNs-PAMA-Pt(IV) possesses a multifunctional platform for trimodal imaging and therapeutic applications (Deng et al., 2015). Further experimental results manifest that such nanocarriers are capable of exerting significant therapeutic efficacy in cancer. In conclusion, many research studies have certified that nanomaterials represent excellent and remarkable merits in the therapy of cancer.

Herein, we categorize the diversity of NDDIP based on stimulus and emphasize the most recent treatment research for cancerous diseases. Simultaneously, we discuss the latest application and development trends of NDDIP in the future.

## Characteristics of the Tumor Microenvironment

TME is of crucial effect on cancer generation, progression, invasion, metastasis, and drug resistance. It is composed of CAFs, ECM, mesenchymal cells, blood vessels, and interstitial fluid (Barati Bagherabad et al., 2017; Anderson and Simon, 2020). CAFs, as the main components of the tumor stroma, are mainly located in the perivascular or peripheral interstitial fibers, secreting cytokines and related enzyme molecules. ECM deposition can produce a dense fibrous interstitium that encapsulates the tumor, making the tumor tissue more fragile and stiffer than normal tissue, forming a physical barrier to immune cell infiltration and inhibiting the targeting of antitumor drugs. Meanwhile, CAFs secrete proteases that remodel the ECM and release chemokines, growth factors, and proangiogenic factors, promoting the malignant transformation of tumors (Jiang et al., 2020). With rapid tumor growth and vascular anisotropy, the intratumor blood supply is often inadequate, causing the tumor microenvironment in a long-term hypoxic environment (Wang et al., 2018a; Chamundeeswari et al., 2019). Additionally, aerobic glycolysis significantly enhanced, further aggravating the acidification in the tumor microenvironment. Generally, the extracellular pH of normal tissues fluctuates within an appropriate range of 7.2–7.4. But the pH value in the majority of tumor extracellular sites is typically lower than that of normal tissue, within the scope of 6.2–6.9 (Ma et al., 2018). Therefore, novel pH-sensitive NDDIPs as a target are promising in the therapeutic efficacy of cancer therapy.

Glutathione (GSH), as an antioxidant, is one of the most important enzymatic platforms which keep the intracellular redox balance in cancer cells. Cysteine, glutamic acid, and glycine compose of GSH exist in two interconvertible and active forms: reduced form (GSH) and oxidized form (GSSG). Intracellularly, a large proportion of GSH is existed in the cytosol with more than 90%, while mitochondria exist at nearly 10% and the endoplasmic reticulum contains an extremely small amount (Rozanov et al., 2015). Many malignant tumor cells are especially dependent on elevated GSH. Therefore, the GSH system has attracted the intensive attention of pharmacologists as a hope target for medical intervention against cancer progression (Nunes and Serpa, 2018; Marengo et al., 2019).

ROS, as byproducts of incomplete aerobic respiration, refer to a series of the highly reactive substances, existing in two forms of radical and nonradical, which include hydrogen peroxide (H_2_O_2_), singlet oxygen (^1^O_2_), peroxide (O_2_^2-^), superoxide (O_2_^−^), and hydroxyl radical (•OH) (Sai et al., 2021; Zhu et al., 2022). ROS functions as a double-edged sword *in vivo* (Zhao et al., 2022; Chen et al., 2021). Some research confirmed that ROS has the capability of stimulating physiological cellular proliferation, and the substances can be utilized by a tumor to promote survival and differentiation. However, high levels of ROS can have deleterious effects, breaking down lipids, proteins, and genetic material DNA. Furthermore, many research studies have confirmed that ROS regarded as an effective tumor inhibitor to achieve potent cancer treatment (Ming et al., 2020; Malla et al., 2021). In conclusion, concentrations of ROS in tumor tissues and intracellular cancerous cells are enormously higher in comparison with normal cells (Weinberg et al., 2019).

## Nanoscale Drug Delivery and Imaging Platform

NDDIP is one of the most spectacular and significant prospects in the targeted drug delivery therapeutics and efficacy evaluation agents (Aminu et al., 2020). It has been used to deliver multitudinous chemotherapeutic drugs, inhibitors, vaccines, and proteins. Among them, the most tremendous promise of nanotechnology is NDDIP complexes.

**FIGURE 1:**
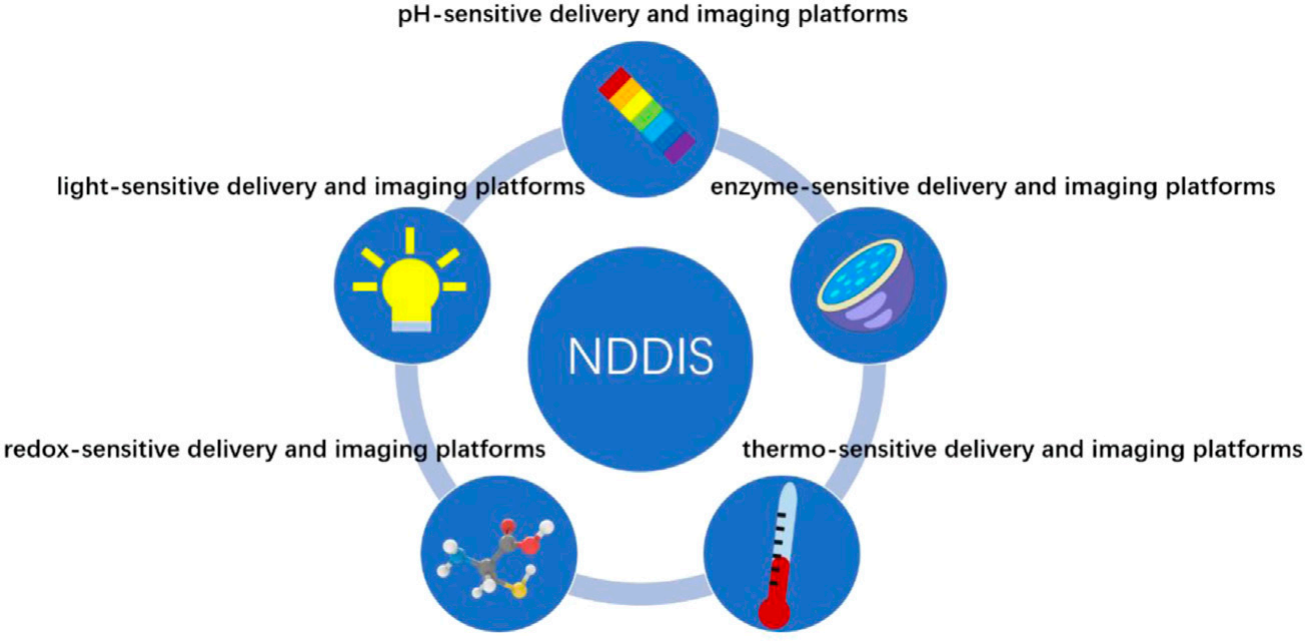
Graphical summarization of five different types of stimuli put into the design of stimuli-sensitive nanocarriers for drug delivery and imaging platforms: pH-sensitive delivery and imaging platforms, enzyme-sensitive delivery and imaging platforms, thermo-sensitive delivery and imaging platforms, redox-sensitive delivery and imaging platforms, and light-sensitive delivery and imaging platforms.

Smart NDDIP combined with stimuli-sensitive properties that can either respond to physical or biological stimuli has recently become the center of attention. According to special characteristics of the diseased microenvironment, NDDIP for drug delivery and imaging platforms can be classified into pH-sensitive delivery and imaging platforms, enzyme-sensitive delivery and imaging platforms, thermo-sensitive delivery and imaging platforms, redox-sensitive delivery and imaging platforms, and light-sensitive delivery and imaging platforms as seen in Table 1 and Figure 1.

**TABLE 1:** Illustrative examples of NDDIP and their biomedical applications.

Nanomaterials platform	Type	Imaging	Application	Characteristic	Ref
Mesoporous silica nanoparticle	pH-sensitive	T_1_-weighted MRI	Tumor tissue	The acetal at acidic pH could generate physicochemical property variations to the removal of the S-NPs to release DOX in the cancer sites	Chen et al. (2015)
HER-DMNPs	pH-sensitive	T_2_-weighted MRI	Tumor tissue	The π–π interactions between DOX and pyrene groups could be decreased at acidic pH to release DOX	Lim et al. (2011)
Poly[N-(2-hydroxypropyl)methacrylamide] (polyHPMA) copolymer	Enzyme- and pH-sensitive	—	Breast cancer	The conformational changes of HPMA take place in DOX releasing from the conjugate under the high concentration of cathepsin B and lower pH value	Wei et al. (2016)
Gelatin nanoparticle	Enzyme-sensitive	Fluorescence imaging	Tumor tissue	Gelatin NPs could be degraded when exposed to a higher concentration of matrix metalloproteinase-2 enzymes in the TME, achieved PAMAM dendrimers release, and improved intracellular uptake into tumor cells	Fan et al. (2017)
Fe_3_O_4_@P(MEO_2_MA_60_-OEGMA_40_)NP	Thermo-sensitive	—	Tumor tissue	The drug release is accelerated based on the high temperature which is raised above the CST, inducing polymer chains to shrink to release DOX.	Jamal Al Dine et al. (2017)
M-MNCs	pH- and thermo-sensitive	T_2_-weighted MRI	Lung cancer cell	M-MNCs possessed pH- and thermo-sensitive release behavior in which the released amount was highest	Farshbaf et al. (2018)
mPEG-ss-Tripp	Redox-sensitive	AIE imaging	Tumor tissue	Disulfide bonds are sensitive to reductive substance GSH, which quickly cleaved through thiol-disulfide exchange to release DOX	Sun et al. (2021)
DOX@MSN-ss-GHA	Redox-sensitive	T_1_-weighted MRI	Tumor tissue	A higher concentration of GSH prompted the disulfide linkage in MSN-ss-GHA to cleave occurring in the cancer cellular environment which achieved drug efflux rapidly	Chen et al. (2016)
FCCP NPs (Fe3O4@CS/CuS NPs)	Redox-sensitive	T_2_-weighted MRI	Tumor tissue	The generation of •OH as a therapeutic agent and the concurrent production of O_2_ as a conditioning agent for cancer theranostics	Zhang et al. (2018b)
AuNSt-MSNP	Light-sensitive	—	Tumor tissue	Thiolated photolabile molecule is sensitive to the NIR light, leading to the formation of succinic acid which triggered controlled DOX-specific release based on irradiation with NIR-light	Hernández Montoto et al. (2019)
Multifunctional anticancer-drug carrier	Light-sensitive	T_2_-weighted MRI	Tumor tissue	The temperature increases from laser irradiation for precise control of the DOX release	Yoon et al. (2019)

### pH-Sensitive Delivery and Imaging Platforms

In the midst of the various sensitive nanoscale drug delivery platforms, pH-sensitive delivery and imaging platform is known as the most comprehensive strategy as a result of the acidification in the solid tumor microenvironment obviously. It is known that the pH value of the tumor extracellular microenvironment is approximately 6.5, lower than that of normal tissue. Therefore, pH-sensitive delivery and imaging platforms are capable of generating physicochemical property variations at a mild acidic tumor microenvironment for their structure or conformation characteristics, resulting in anticancer drugs or contrast enhancement at the desired site. Then, multiple imaging methods were used to observe the changes in lesion size and evaluate the therapeutic effect of the tumor (Yang et al., 2019).

On account of this sensitive reaction, Chen et al. developed a multifunctional mesoporous silica nanoparticle system for drug delivery and magnetic resonance imaging (MRI) by a classic method (Chen et al., 2015) (see Table 1). The pH-responsive MS@S-NPs are the essential component of the mesoporous silica nanoparticle system. DOX hermetically loaded into the holes of MS, and then the holes were sealed with S-NP to prevent premature drug release. As the experiment proves, MS@S-NPs were stable under conditions similar to physiological status (pH = 7.4), and the DOX release generally ignored. On the contrary, the DOX release markedly increased with the reduction of pH. This is principally because the acid–labile acetal bridges the gaps between MS and S-NPs, and the hydrolysis of the acetal at acidic pH as triggers could generate physicochemical property variations to remove the S-NPs achieving the DOX specific-targeting and controlled release. Due to the presence of Gd^3+^, MS@S-NPs, as an efficient contrast agent, realized T_1_-weighted MR images successfully at the tumor site for image-guided drug delivery and efficacy assessment. Hence, MS@S-NPs, as high effective NDDIP, play important roles in tumor therapy and real-time monitoring (Hrubý et al., 2005; Devalapally et al., 2007).

On this point, a new study has claimed that Lim et al. (see Table 1) designed HER-DMNPs as pH-triggered NDDIP through employing α-pyrenyl-ω-carboxyl poly(ethylene glycol) (Py-PEG-COOH or pyrenyl-PEG) to encapsulate magnetic resonance (MR)-sensitive MnFe_2_O_4_ nanocrystals (MNCs) and doxorubicin (DOX). In this research, the authors demonstrated that the π–π interactions between DOX and pyrene groups could be decreased at acidic pH to release DOX. Meanwhile, the loaded DOX could be effectively conveyed to tumor tissues reducing toxicity to normal tissues, which realizes targeted cancer therapy. In addition, the authors verified that the MnFe_2_O_4_ nanocrystals possessed T_2_-weighted MR imaging capability through experiments that could be monitored by MRI to guide personalized cancer regimens. In summary, HER-DMNPs, as high effective nanoparticles, have a good T_2_-weighted MRI imaging effect for tumor therapeutic effect evaluation (Lim et al., 2011).

### Enzyme-Sensitive Delivery and Imaging Platforms

Enzymes often play pivotal roles in metabolism and their intimate connection with human health greatly. However, there are several enzymes with specific chemical properties overexpressed in the tumor microenvironment, such as hydrolases, cathepsins, kinases, esterases, and gelatinase (Lou et al., 2021; Dhaval et al., 2022; Wang et al., 2018b; Zhang et al., 2018a). Therefore, overexpression of dysregulated enzyme as a favorable potential biological target is of new innovations. In brief, smart NDDIP stimulated by enzymes has a tremendous and excellent foreground to develop as one of the promising cancer therapies (Chau and Zhong, 2011; Rastegari et al., 2017).

Cathepsin B overexpression has been related to more premalignant lesions playing an important role in adjusting the tumor microenvironment in a wide variety of cancer types. Hence, the cathepsin B-sensitive delivery platform has been conceived, using enzyme as triggers, to deliver the drug to the targeted diseased site where the cathepsin B is overexpressed. Wei and coworkers developed functional polyHPMA copolymer-loaded DOX as enzyme- and pH-sensitive delivery platforms with favorable promising efficiency for breast cancer therapy (Wei et al., 2016) (see Table 1). HPMA was prepared by RAFT polymerization, possessing cathepsin B sensitive moiety (GFLGK) loaded to branched copolymers. Then, obtained copolymer covalently bound to the DOX via pH-sensitive hydrazine bond. In this study, an enzyme complex based on cathepsin B could develop rapid and efficient cleavage. Moreover, the DOX release lies on the pH of the tumor environment. Therefore, the conformational changes of HPMA take place in DOX releasing from the conjugate under a higher concentration of cathepsin B and lower pH value. The novel HPMA copolymer had successfully applied to 4T1 tumor models with enhanced antitumor efficacies. In conclusion, the enzyme-sensitive delivery platforms are regarded as a safe and high effective nanoparticle for breast cancer therapy.

Fan et al. designed gelatin nanoparticles containing PAMAM dendrimer-loaded MTX to improve antitumor efficiency (see Table 1). The high concentration of matrix metalloproteinase-2 enzymes in TME effectively promoted gelatin nanoparticle cracking, which achieved PAMAM dendrimers release and improved intracellular uptake into tumor cells. Meanwhile, the results demonstrate that the multistage nanocarrier guaranteed the therapeutic effect of antitumor drugs. Nanocarriers have ascendancy in fluorescence imaging to assess drug distribution in real time (Fan et al., 2017).

### Thermo-Sensitive Delivery Platforms

Thermal is one of the most-explored external stimuli-sensitive for NDDIP, which is delicately sensitive to the thermal within an imperceptible transformation, especially in cancer therapy and diagnosis. The normal body temperature is about 37°C, while the temperature rises in the existence of pyrogens or pathogens, diverging from the normal temperature, which can be exploited to motivate the release of active ingredient pharmaceutical compounds for thermo-sensitive drug delivery platforms. Favorable thermo-sensitive delivery platforms stably reserve therapeutic drugs at physiological temperatures, and they merely released them to the targeted diseased site where the temperature is higher than the critical temperature of drugs. In addition, admired NDDIPs possessed favorable biocompatible and well-stable loading capacity (Qian et al., 2013; Limjeerajarus et al., 2020).

Recent research by Enaam et al. demonstrated that Fe_3_O_4_@P(MEO_2_MA_60_-OEGMA_40_)NP is equipped with reversible aggregation in response to the temperature used to make a targeting drug-carried platform (Jamal Al Dine et al., 2017). (see Table 1). Fe_3_O_4_@P(MEO_2_MA_60_-OEGMA_40_)NP, as targeted drug carriers, was composited by a mild method, and then the nanoparticles encapsulated DOX by noncovalent interactions bounding to thermo-responsive polymers which possessed a critical temperature of around 41°C. The properties of DOX drug release increase from the nanocarriers above 40°C was proved by a series of experimental investigations. It was found that the drug release efficiency of the nanocarriers might be concerned with thermo-responsive polymers. The main cause for the release of DOX is that the polymer chains shrunk when the temperature is higher than the critical temperature. In brief, the favorable thermo-sensitive delivery platforms of Fe_3_O_4_@P (MEO_2_MA_60_-OEGMA_40_)NP with precisely regulated might provide great significance and potential for drug delivery.

Masoud and coworkers synthesized multifunctional pH- and thermo-sensitive delivery platforms (M-MNCs) through a multistep, and then mesoporous silica successfully connected to polymers as an anticancer drug payload to realize release of loaded medicine in specific circumstances (see Table 1). The authors prove by experiment that the M-MNCs showed pH- and thermo-sensitive release behavior which the released amount was highest (92.3%) at pH 5 and 40°C. Therefore, the multifunctional delivery platforms have excellent capability in drug loading and releasing. Besides, the study results showed that M-MNCs retained superparamagnetic characteristics as promising T_2_-weighted contrast agents to evaluate the therapeutic effect (Farshbaf et al., 2018).

### Redox-Sensitive Delivery and Imaging Platforms

Redox-sensitive delivery platforms have moved into the spotlight based on the high concentrations of redox agents such as ROS and GSH in the solid tumor microenvironment, compared with their concentration in normal sites sharply. It has extensively enormous potential utilized as favorable potential triggers to controlled drug-specific release contenting in response to intracellular conditions for cancer therapeutic strategy without damaging the other normal sites surrounding the tumor mass. Herein, redox-responsive NDDIPs are promising in realizing efficient intracellular stimulus-responsive drug platforms (Zhao et al., 2016).

**FIGURE 2:**
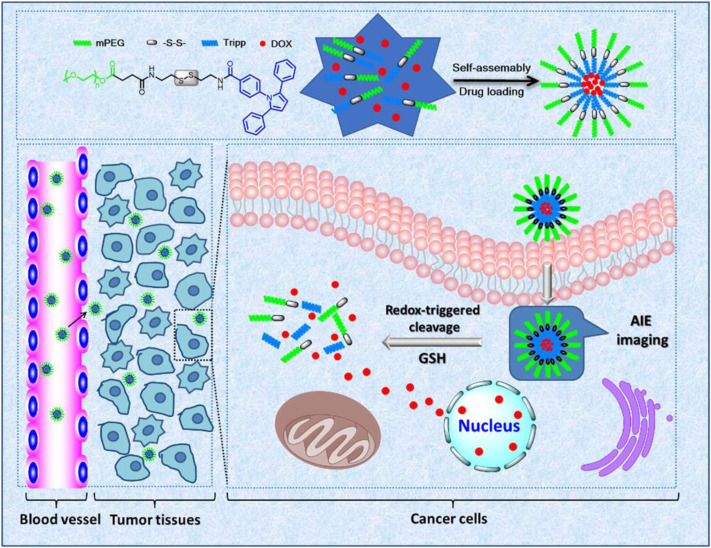
**|** Malignant tumor cells are characterized by intracellular high concentrations of GSH. Advantage is taken from this in developing the redox-responsive drug delivery platform. Sun and colleagues explore redox-sensitive delivery platform (mPEG-ss-Tripp). Synthesized mPEG-ss-Tripp was loaded with doxorubicin via a disulfide bond, which is sensitive to reductive substance GSH and then quickly cleaved through thiol-disulfide exchange, in order to release the DOX at targeted sites without damaging normal tissues (Sun et al., 2021).

As is known to all, the GSH concentration in tumor cells is typically higher than the values of healthy cells (Mirhadi et al., 2020). Sun and colleagues took advantage of this characteristic to explore a redox-sensitive delivery platform (mPEG-ss-Tripp) used for controlled drug delivery and simultaneous AIE imaging (Sun et al., 2021) (see Table 1). By the self-assembled method, mPEG-ss-Tripp was synthesized and simultaneously loaded doxorubicin via a disulfide bond. Disulfide bonds are sensitive to reductive substance GSH, which is quickly cleaved through thiol-disulfide exchange, especially in the cancer intracellular environment under a higher concentration of GSH. On the contrary, disulfide bonds have fixed construction in normal tissues at a lower level of GSH. *In vitro* cytotoxicity of micelles experiments proved that the toxicity of DOX-loaded micelles to 293 T cells is extremely low. However, DOX-loaded micelles can prevent 4T1 cell growth and show great cell toxicity. Therefore, mPEG-ss-Tripp has good tumor selectivity between cancer cells and normal cells. At the same time, the mPEG-ss-Tripp with AIE property achieved cellular imaging for monitoring the distribution of the drug as seen in Figure 2.

Chen et al. explored an effective NDDIP for multipurpose cancer theranostics (see Table 1). Obtained MSN-ss-GHA could not only achieve medicine transportation under a strong reducing condition but also dramatically improve 4T1 intracellular uptake. With the assistance of an ultrasonic bath, anticancer drugs were successfully loaded into MSN-SS-GHA to further synthesize DOX@MSN-ss-GHA. A higher concentration of GSH prompted the disulfide linkage in MSN-ss-GHA to cleave occurring in the cancer cellular environment which achieved anticancer drug efflux rapidly. Moreover, the presence of Gd component is the basis of excellent T_1_-weighted MRI imaging. In conclusion, the new redox-sensitive delivery platforms with accurately cleaved disulfide linkage revealed enormous meaningful and promising for neoadjuvant chemotherapy. At the same time, redox-sensitive delivery and imaging platforms are regarded as high effective nanoparticle in tumor precision therapy and real-time monitoring by MRI. (Chen et al., 2016).

Zhang et al. synthesized FCCP NPs (Fe_3_O_4_@CS/CuS NPs) nanoclusters to achieve multimodal imaging and synergetic therapy (see Table 1). As obtained, Fe_3_O_4_@CS/CuS NPs performed important functions in improving intrinsic peroxidase to generate •OH and O_2_ from endogenous H_2_O_2_, in which the generation of •OH as a therapeutic agent suppresses the cancerous cells and the concurrent production of O_2_ as a conditioning agent modulates the hypoxic microenvironment of tumors with remarkable efficiency. In conclusion, ROS is regarded as an effective tumor inhibitor to achieve potent cancer treatment. Due to the presence of Fe, the nanoparticle is a potential T_2_ contrast agent for MRI which is proportionate to the increase of the Fe concentration. *In vivo* and *in vitro* experiments proved that FCCP NPs inhibiting tumor growth were remarkably improved. T_2_-weighted MR imaging was achieved to effectively evaluate the targeting of nanoparticles. In summary, FCCP NPs could apply in the precise diagnosis and efficient therapeutic and are expected to contribute to personalized medicine (Zhang et al., 2018b).

### Light-Sensitive Delivery and Imaging Platforms

Innocuous electromagnetic radiation contained ultraviolet (UV), visible light (VIS), and near-infrared light (NIR), as light-sensitiveness stimuli have moved into the considerable spotlight due to favorable biocompatibility and tissue penetration. Novel light-sensitive delivery platforms based on electromagnetic radiation with an external light source have been recently exploited to achieve more drug delivery at well-delimited sites of the body, guaranteed selective drug accumulation and reduced drug side effects, and improved pharmacokinetics and pharmacodynamics, to realize secure and efficient cancer therapy. Taking advantage of light-sensitive delivery platforms, external stimuli can be used to develop light-sensitive delivery platforms to improve a range of applications.

At present, multifarious NIR-sensitive delivery platforms using different responsive mechanisms have been generated and investigated for the treatment of cancer (Chamundeeswari et al., 2018).

In a recent study, Andy and colleagues investigated the NIR-light-triggered drug delivery approach (Hernández Montoto et al., 2019) (see Table 1). AuNSt with a thiolated photolabile molecule was firstly acquired via a simple and mild method, and then the thiol groups were connected AuNSt and MSNP@DOX together in order to synthesize the satisfying NIR-sensitive drug delivery. The principle of AuNSt-MSNP is that thiolated photolabile molecule is sensitive to the NIR light leading to the formation of succinic acid which is triggered to controlled DOX specific release based on irradiation with NIR-light. *In vitro* cytotoxicity of AuNSt-MSNP experiments proved that DOX-loaded nanoparticles revealed nontoxicity to HeLa cells without NIR light, while under NIR laser, AuNSt-MSNP severely inhibited HeLa cells viability by effectively releasing DOX. In conclusion, this novel NIR-light-triggered drug delivery platform has provided a promising potential for achieving the synchronism of treatment to strengthen the efficiency of combination therapy (Xiang and Chen, 2020).

In another study, Yoon et al. have developed a multifunctional anticancer-drug carrier containing GNR, IONP, and DOX, with a desirable small size (see Table 1). GNR is efficiently able to transform light into heat energy under NIR. IONP serves as an MRI contrast agent monitoring with the MRI T_2_ imaging precisely. Experiments have proved that the temperature increase from laser irradiation to achieve drug release precisely, reducing damage to normal surrounding tissue. Meanwhile, the workers proved the property of drug release *via* MTT which utilized HeLa cells as a stable cell model. The result reflects that DOX is released from the multifunctional anticancer-drug carrier by laser stimulation to effectively kill cancer cells. In conclusion, the multifunctional light-sensitive delivery and imaging platforms could not only resoundingly release DOX into the cancer cells under NIR but also achieve MRI T_2_ imaging (Yoon et al., 2019).

## Future Outlooks

With the therapeutic strategy of carcinoma flourishing, and life-span lengthening, the solicitude for the patients’ quality of life appears to be more significantly momentous. The current modalities of traditional cancer therapies comprise surgery, chemotherapy, radiotherapy, and their combinations and is extremely effective to make tumor shrink or disappear, simultaneously inevitably damaging the other normal sites surrounding the tumor mass. Therefore, probing into how to suppress tumor growth and prolong the survival, while simultaneously not injuring the human organism, is a crucial and aggressive cancer treatment protocol. Nanomedicine, especially NDDIP, has recently promoted immense and innovative preclinical and utility progress in health care. NDDIP as efficient and favorable drug delivery and diagnostic tools would witness deep involvement of various diseases, with targeted and stimuli-responsive ability to deliver drugs. Synchronously, it has solved side effects caused by the traditional therapeutic strategies of carcinoma and achieved the development of precision medicine *via* reduced systemic cytotoxicity, improved cancerous tissue permeability, and prolonged circulation time.

As we have evidenced by the studies in this review, the performance of NDDIP is verified via substantial preclinical studies, which possesses promising efficiency to suppress the growing progress of cancer (Wu et al., 2016). In this field, plenty of nanoparticles, possessing the characteristics of responding to pH, enzyme, thermal, redox, and light, have been prepared and developed to achieve precise and targeted therapy. Not long afterward, abundant and varied NDDIPs with specific tumor targeting and on-demand antitumor efficiency will appear soon in the clinical application (Zhao et al., 2018).

In conclusion, scientists continuously look for opportunities to improve favorable and profound ways to treat many kinds of cancer by optimizing and innovating nanoparticles and technologies. Therefore, the appearance of smart NDDIP platforms would enable to perform therapy and diagnostics of oncology patients at a totally new and advanced level, maximizing the therapeutic effects, and improving the patients’ quality of life.
